# Shift Work and Psychological Distress Among Healthcare Workers: The Mediating Role of Sleep Quality and the Association with Job Dissatisfaction

**DOI:** 10.3390/healthcare14152372

**Published:** 2026-08-03

**Authors:** Furkan Bakırhan, Osman Küçükkelepçe

**Affiliations:** 1 Faculty of Medicine, Adıyaman University, Adıyaman 02040, Türkiye; furkan.bakirhan44@gmail.com; 2 Gülhane Medical Faculty, University of Health Sciences, Ankara 06800, Türkiye

**Keywords:** shift work, sleep quality, psychological distress, DASS-21, PSQI, job satisfaction, healthcare workers

## Abstract

**Background/Objectives**: The study evaluated the associations among shift work, sleep quality, psychological distress, and job dissatisfaction among healthcare workers and examined the indirect association between shift work and psychological distress through sleep quality. **Methods**: This cross-sectional analytical study included 276 healthcare workers at Adıyaman Kahta State Hospital. Sociodemographic and occupational characteristics were assessed using a questionnaire; psychological distress was measured with the Depression Anxiety Stress Scale-21 (DASS-21), and sleep quality with the Pittsburgh Sleep Quality Index (PSQI). Associations were examined using regression and mediation analyses. **Results**: Overall, 51.1% of participants worked shifts, and 46.0% reported job dissatisfaction. Shift workers had higher PSQI scores than regular daytime workers (10.21 ± 3.22 vs. 7.90 ± 3.48; *p* < 0.001) and higher DASS total scores (25.16 ± 9.35 vs. 20.28 ± 13.43; *p* < 0.001). PSQI scores were positively correlated with depression, anxiety, and stress scores. The mediation analysis showed a statistically significant indirect association between shift work and psychological distress through poorer sleep quality; the direct association was not statistically significant after sleep quality was included in the model. Psychological distress was independently associated with job dissatisfaction. **Conclusions**: These findings suggest that sleep quality may represent a statistical intermediary in the association between shift work and psychological distress. Sleep-focused and organisational strategies may warrant evaluation as potential approaches to support healthcare worker well-being.

## 1. Introduction

Shift work refers to a broad range of work schedules performed outside traditional daytime working hours, including occasional overnight on-call duties, rotating schedules, permanent night shifts, and work patterns that disrupt normal nighttime sleep [1,2]. Approximately one-fifth of the global workforce is engaged in shift work, and the healthcare sector is among the sectors in which shift work is most common, followed by transportation and industry [3,4,5]. In healthcare settings, shift work is necessary to ensure continuity of care and is often organised through fixed or rotating schedules. However, both types of schedules may disrupt circadian rhythms that regulate vital physiological functions, thereby affecting the health and quality of life of healthcare workers [5,6,7]. Circadian disruption may impair the functioning of several biological systems and contribute to adverse outcomes, including metabolic disorders, cardiovascular and mental health problems, and cancer-related risks. In addition to its contribution to various health problems, circadian disruption may also be associated with increased workplace errors and poorer work-related outcomes [2,6,8].

Sleep quality is an important determinant of quality of life and perceived health status [7]. Shift workers often experience fatigue and disruption of the sleep–wake cycle because daytime sleep does not provide the same restorative effects as nighttime sleep [9]. Shift work and night work may also lead to sleep interruption, poor sleep quality, and daytime sleepiness [7]. In addition, impaired sleep quality has been associated with a greater tendency toward depressive and anxiety symptoms [3,10,11]. Sleep disturbances affect not only circadian patterns but also mood, perception, memory, stress response, and daily functioning [7,12]. They may lead to sleepiness and chronic fatigue, thereby impairing cognitive function, alertness, and work performance [3,9,10]. Sleep deprivation itself has been associated with cardiovascular morbidity, metabolic syndrome, psychosocial problems, and reduced overall well-being [13,14,15].

Although shift work is necessary for the continuity of healthcare services, it may have adverse physical and psychological consequences by disrupting circadian and homeostatic rhythms [7,16,17,18]. This work pattern has been associated with sleep problems, fatigue, stress, reduced job satisfaction, and disruption of social life; potential effects on metabolic, cardiovascular, reproductive, and cancer-related outcomes have also been reported [7,19,20,21,22]. Poor sleep quality may represent a potential explanatory pathway in the association between shift work and psychological distress. This proposed pathway can be understood through both biological and psychological mechanisms. Night work may disrupt the alignment between circadian rhythms and work–rest schedules, leading to alterations in melatonin secretion, hypothalamic–pituitary–adrenal axis activity, autonomic nervous system balance, and inflammatory responses. These processes are closely involved in stress reactivity and emotional regulation. Insufficient or poor-quality sleep may also reduce the restorative benefits of sleep and weaken prefrontal regulatory control, potentially increasing vulnerability to anxiety, depressive symptoms, irritability, and perceived stress [23]. From a psychological perspective, sleep impairment may compromise coping capacity, cognitive flexibility, attention, and recovery from occupational stressors. In healthcare settings characterised by heavy workloads and night shifts, these effects may further increase workers’ vulnerability to psychological distress. Consistent with this proposed pathway, a recent study among emergency nurses found that night-shift work was associated with shorter sleep duration, impaired prefrontal cerebral oxygenation, poorer cognitive performance, and higher state anxiety [24]. Night shift work may not only disrupt sleep patterns but also increase the risk of major traffic accidents or near-accident experiences due to sleepiness while driving, reduced vehicle control, and difficulty staying in a lane [6,25]. It may also impair decision-making, increase the likelihood of accidents both at and outside work, and reduce efficiency and productivity. Furthermore, sleepiness and fatigue related to shift work may increase the risk of medical errors, reduced quality of healthcare services, compromised patient safety, and occupational injuries [1,9,26,27].

The psychological well-being of healthcare workers is critical for delivering high-quality patient care. Due to the demanding, stressful nature of healthcare work and the challenges of night work, healthcare workers may experience increased stress, burnout, and reduced job satisfaction. Continuous exposure to such unfavourable working conditions may also contribute to the development of mental health problems, including depressive and anxiety symptoms [6,28,29]. Some workers may use or misuse different substances, such as over-the-counter medications, alcohol, khat, or tobacco, as a way of coping with stress related to shift work [1,30]. Shift work has also been associated with substance use or misuse among nurses [1,31,32].

Existing literature indicates that shift work may impair sleep quality, increase psychological symptoms, and reduce job satisfaction among healthcare workers. Job dissatisfaction is an important occupational health outcome that may affect healthcare workers’ professional well-being, organisational commitment, work performance, and quality of care. In healthcare settings, heavy workloads, night shifts, irregular working hours, high levels of responsibility, and limited recovery time may increase workers’ psychological burden. Stress, anxiety, depressive symptoms, and burnout may, in turn, adversely affect how workers perceive their jobs and their level of job satisfaction [33]. Accordingly, examining the association between psychological distress and job dissatisfaction may provide a more comprehensive understanding of how shift work is associated with occupational well-being. A recent systematic review and meta-analysis reported an inverse association between job satisfaction and burnout among intensive care unit nurses. They identified several relevant organisational factors, including professional experience, working conditions, the work environment, salary, length of service, and access to mental health support [34]. These findings support considering job dissatisfaction alongside shift work, sleep quality, and psychological distress within an integrated occupational health framework. However, most previous studies have evaluated these outcomes separately, and the possible mediating role of sleep quality in the association between shift work and psychological distress, as well as the relationship between psychological distress and job dissatisfaction, have not been sufficiently examined within a single analytical framework. This gap is particularly important in healthcare settings, where long and irregular duty hours are common, and the pathways underlying the associations between shift work and employee well-being remain insufficiently understood. Therefore, this study compared the sociodemographic and occupational characteristics, depressive symptoms, anxiety symptoms, stress symptoms, and sleep quality of healthcare workers employed under regular daytime and shift work schedules. It also evaluated the association of shift work with psychological distress and job satisfaction. In addition, the study examined whether the data were consistent with a statistical indirect association between shift work and psychological distress through sleep quality, as well as the association between psychological distress and job dissatisfaction, within an integrated mediation-and-outcome model.

## 2. Materials and Methods

### 2.1. Study Design and Population

This analytical cross-sectional study examined the relationships among shift work, sleep quality, psychological distress, and job satisfaction among healthcare workers. Data were collected between 15 March and 15 June 2025.

Kahta State Hospital was purposively selected for feasibility because the research team had an established collaboration with the institution, and the hospital administration agreed to facilitate data collection. In addition, the relevant workforce was considered sufficiently large to achieve the target sample size. At the time of the study, the hospital employed 275 nurses and approximately 150 medical secretaries and data entry personnel, yielding an estimated source workforce of 425 employees in the occupational groups targeted for recruitment.

The study population comprised nurses, medical secretaries, and data entry personnel working at Kahta State Hospital. Medical secretaries and data entry personnel were defined as staff members who supported clinical service processes in outpatient clinics, intensive care units, and inpatient wards by performing tasks such as patient registration, admission and discharge procedures, and entering patient information into the hospital information system. Participants were recruited from shift-based units, including intensive care units and inpatient wards, as well as from outpatient clinics and administrative units operating during regular daytime hours.

Employees in the specified occupational groups who had worked at the hospital for at least six months were eligible for inclusion. Student interns and employees with a previously diagnosed sleep disorder, anxiety disorder, or depressive disorder were excluded.

### 2.2. Sample Selection

Participation in the study was voluntary. The minimum target sample size was estimated using the single-population proportion formula, n = Z^2^ × p(1 − p)/d^2^. A previous study reporting a night-shift work prevalence of 77% among healthcare workers was used to determine the expected proportion (*p* = 0.77) [5]. With a 95% confidence level (Z = 1.96) and a 5% margin of error, the minimum target sample size was calculated as 273 participants. A finite population correction was not applied, thereby maintaining a conservative sample-size target despite the estimated source workforce of 425 employees. To account for potential nonresponse and incomplete questionnaires, the recruitment target was increased by approximately 10% to 300 participants.

A non-probability convenience sampling method was used. During the recruitment process, information obtained from the hospital administration indicated that relatively few physicians worked under the shift arrangements examined in this study and that their overall number was considerably smaller than the numbers of employees in the occupational groups targeted for recruitment. Physicians were therefore not approached for participation, and recruitment focused on nurses, medical secretaries, and data entry personnel, from whom approximately comparable shift-work and regular daytime-work groups could feasibly be formed. Because employees working rotating or night schedules were not always present or accessible during the data collection period, eligible personnel who were available were invited to participate. Recruitment was monitored to obtain approximately comparable numbers in the two work-schedule groups. Overall, 387 employees were approached, of whom 301 agreed to participate (77.8%). Twenty-five questionnaires were excluded because of incomplete responses, resulting in a final analytic sample of 276 participants, corresponding to 71.3% of all employees approached.

### 2.3. Data Collection Tools

Data were collected using a questionnaire consisting of sociodemographic data, the Depression Anxiety Stress Scale-21 (DASS-21), and the Pittsburgh Sleep Quality Index (PSQI).

A structured information form was used to collect participants’ sociodemographic, occupational, health-related, and lifestyle characteristics. The form included questions on age, sex, educational status, marital status, height and weight, number of children, perceived economic status, occupation, work unit, years of professional experience, overtime work, shift-work status, presence of chronic disease, mood status, tobacco and alcohol use, daily consumption of caffeinated beverages, mode of transportation to work, sleepiness when leaving work, near-accident experiences and their types, daily sleep duration, job satisfaction, and reasons for job dissatisfaction.

Job satisfaction was assessed using a single self-reported item. Participants were asked whether they were satisfied with their current job, with response options of “yes” and “no.” For statistical analyses, participants who responded “yes” were classified as job satisfied, whereas those who responded “no” were classified as job dissatisfied. Participants reporting job dissatisfaction were additionally asked to select the main reason for their dissatisfaction from a set of predefined response options. These categories were: (1) emotional burden and interpersonal problems, (2) financial difficulties, (3) problems related to work schedules, colleagues, or time constraints, and (4) health problems. In addition, participants were asked to rate their intention to resign from their job on a scale from 1 to 10, where 1 indicated “I do not think about resigning at all” and 10 indicated “I definitely think about resigning.” Similarly, perceived health status was assessed on a scale from 1 to 10, where 1 indicated “unhealthy” and 10 indicated “healthy.”

Intention to leave was assessed using a single self-reported item. Participants were asked to rate their intention to leave their current job on a numerical scale ranging from 1 to 10, with higher scores indicating a stronger intention to leave.

The DASS-21 was developed by Lovibond and Lovibond in 1995 [35]. In the present study, the Turkish adaptation developed by Sarıçam in 2018 was used [36]. The scale consists of 21 items, three subscales (depression, anxiety, and stress), and a four-point Likert-type scoring system ranging from 0 (“never”) to 3 (“always”). Item scores within each subscale are summed to obtain the total score for that subscale. Higher subscale scores indicate greater levels of depressive symptoms, anxiety symptoms, and stress symptoms. The Cronbach’s alpha internal consistency coefficients reported for the depression, anxiety, and stress subscales were 0.87, 0.85, and 0.81, respectively. In the present study, Cronbach’s alpha values were 0.842 for the stress subscale, 0.843 for the anxiety subscale, and 0.881 for the depression subscale.

The Pittsburgh Sleep Quality Index (PSQI) was developed by Buysse et al. in 1989 [37]. The Turkish adaptation was conducted by Ağargün et al. in 1996 [38]. The PSQI consists of 19 items and is designed to assess sleep disturbances and sleep habits over the previous month. Each component is scored from 0 to 3, and the sum of the component scores yields the total PSQI score. The total score ranges from 0 to 21, with higher scores indicating poorer sleep quality. The Cronbach’s alpha internal consistency coefficient of the Turkish version was reported as 0.804. In the present study, Cronbach’s alpha was 0.709.

### 2.4. Ethical Approval

Ethical approval for the study was obtained from the Non-Interventional Research Ethics Committee of Fırat University on 19 December 2024, at meeting number 15–49, with decision number 29991. Written permission was also obtained from the hospital administration to conduct the study. The study was conducted in accordance with the ethical principles of the Declaration of Helsinki. Written informed consent was obtained from all participants. Participation in the survey was voluntary, and participants were informed that they could refuse to participate or withdraw at any time without penalty. Participant anonymity and confidentiality were strictly maintained. Electronic data were stored and encrypted on a password-protected computer. Only the researcher had access to the survey forms and digital copies of the electronic data.

### 2.5. Statistical Analysis

The data were entered into IBM SPSS Statistics for Windows, Version 27.0, and data cleaning, error checking, descriptive tables, and statistical analyses were performed using this software. Categorical variables were presented as numbers (*n*) and percentages (%). Continuous variables were expressed as mean ± standard deviation and/or median (minimum–maximum), depending on their distributional characteristics.

The normality of continuous variables was assessed using the Kolmogorov–Smirnov and Shapiro–Wilk tests, histograms, and Q–Q plots. For normally distributed continuous variables, Student’s *t*-test was used for comparisons between two groups, and one-way analysis of variance (ANOVA) was used for comparisons among three or more groups. For non-normally distributed continuous variables, the Mann–Whitney U test was used for comparisons between two groups, and the Kruskal–Wallis H test was used for comparisons among three or more groups. Associations between categorical variables were evaluated using Pearson’s chi-square test. When expected cell counts were insufficient, Fisher’s exact test or, where appropriate, Monte Carlo-corrected chi-square tests were used.

The relationships between the DASS-21 subscales and the PSQI total score were evaluated using Pearson correlation analysis. Before multivariable modelling, candidate predictors were identified separately for each outcome through bivariable analyses. Variables with *p* < 0.25 in these analyses were considered eligible for inclusion in the corresponding multivariable model. This threshold was used solely as a preliminary screening criterion and was not interpreted as indicating statistical significance. Variables selected a priori based on theoretical or clinical relevance were also retained for consideration irrespective of their bivariable *p*-values. Candidate variables were subsequently evaluated for multicollinearity, and eligible variables without problematic multicollinearity were entered simultaneously into the multivariable models using the enter method. The bivariable screening results and the rationale for the inclusion or exclusion of each candidate predictor are presented in Supplementary Table S1. Multiple linear regression analyses were performed to identify factors independently associated with PSQI and DASS-21 total scores. Multicollinearity was assessed using correlation coefficients, variance inflation factors (VIFs), and tolerance values. Variables with a correlation coefficient ≥ 0.90, VIF > 10, or tolerance value < 0.20 were excluded from the model.

Job dissatisfaction was used as a binary outcome variable in the multivariable logistic regression analysis. This variable was derived from the single self-reported job satisfaction item, with participants who reported being satisfied coded as “0” and those who reported being dissatisfied coded as “1.” Candidate predictors were selected according to the screening and multicollinearity assessment procedure described above. Logistic regression results were presented as odds ratios (ORs) with 95% confidence intervals (CIs). Model calibration was evaluated using the Hosmer–Lemeshow goodness-of-fit test, with *p* > 0.05 interpreted as indicating no evidence of poor model fit. The model’s classification performance was assessed using the overall correct classification rate.

An integrated mediation and outcome model was established to evaluate the relationships among shift work, sleep quality, psychological distress, and job dissatisfaction. In the first stage, mediation analysis was conducted using Hayes’ PROCESS Macro Model 4 to assess the direct and indirect associations between shift work and psychological distress [39]. In this model, shift work was defined as the independent variable, the PSQI total score as the mediator, and the DASS-21 total score as the dependent variable. The shift work variable was coded as “0” for the regular daytime schedule and “1” for the shift work schedule. An increase in the PSQI score indicated poorer sleep quality, whereas an increase in the DASS-21 total score indicated higher psychological distress.

In the mediation analysis, age, sex, marital status, and presence of chronic disease were included as covariates. These variables were selected a priori for their theoretical and clinical relevance as potential confounders. Age and sex may influence both work schedule allocation and vulnerability to sleep disturbances and psychological distress. Marital status may be related to family responsibilities, social support, recovery opportunities, and psychological well-being. The presence of chronic disease was included because underlying health conditions may affect sleep quality and psychological symptom burden independently of work schedule.

These covariates were included to reduce potential confounding in the estimated associations among shift work, sleep quality, and psychological distress. Variables that could be part of the proposed pathway, such as sleepiness after work, daily sleep duration, PSQI score, DASS-21 score, and job dissatisfaction, were not included as covariates in the mediation model to avoid overadjustment.

In the mediation analysis, total, direct, and indirect effect coefficients were evaluated. The statistical significance of the indirect effect was tested using the bootstrap resampling method, and 95% confidence intervals were calculated based on 5000 bootstrap samples. A bootstrap confidence interval that did not include zero was interpreted as indicating a statistically significant indirect effect. In the model, path a represented the association between shift work and sleep quality; path b, the association between sleep quality and psychological distress; path c, the total effect; and path c′, the direct effect.

In the second stage, multivariable logistic regression analysis was performed to evaluate the association between psychological distress and job dissatisfaction. In this model, the DASS-21 total score was the main independent variable, and job dissatisfaction was the dependent variable. The variables included in the model were determined according to the variable selection and multicollinearity assessment approach described for the general logistic regression analysis.

A *p*-value of <0.05 was considered statistically significant in all analyses.

## 3. Results

A total of 276 healthcare workers were included in the study. The mean age of the participants was 33.46 ± 7.74 years; most participants were female (67.4%), had a bachelor’s degree (84.7%), and were married (59.4%). Of the participants, 73.2% were nurses and 26.8% were medical secretaries or data entry personnel. The most common work units were the emergency department (38.8%), outpatient clinics (26.4%), and inpatient wards (15.6%). Overall, 51.1% of participants worked in a shift work schedule, and the most common shift pattern among shift workers was 24-h duty (78.0%) (Table 1).

The mean BMI was 24.91 ± 3.77 kg/m^2^; 35.5% of participants were overweight, and 8.7% were obese. Chronic disease was reported by 20.3% of participants. Active tobacco use was reported by 29.0%, alcohol use by 6.9%, and consumption of five or more caffeinated beverages per day by 30.1%. Sleepiness after work was reported by 57.6% of participants, and 59.4% reported having experienced at least one near-accident (Table 1).

Regarding sleep patterns, 28.3% of participants reported sleeping less than 6 h per day. Job dissatisfaction was reported by 46.0% of participants. Among participants reporting job dissatisfaction, the most commonly reported reason was emotional burden and interpersonal problems (53.5%). The mean resignation intention score was 4.46 ± 2.93, the mean DASS total score was 22.77 ± 11.76, and the mean PSQI total score was 9.08 ± 3.54 (Table 1).

Participants were compared by work schedule: regular daytime workers (*n* = 135) and shift workers (*n* = 141). The proportion of shift work was significantly higher among participants aged 20–29 years, males, unmarried participants, those without children, nurses, those working in high-intensity clinical units, and those with shorter professional experience (*p* < 0.05). In contrast, no significant differences were found between the groups in terms of educational level, income status, mood status, perceived health status, tobacco use, alcohol use, caffeine consumption, mode of transportation to work, near-accident experience, or type of near-accident (*p* > 0.05) (Table 2).

Sleepiness after work, job dissatisfaction, and intention to resign were significantly higher in the shift-work group than in the regular daytime-schedule group (*p* < 0.001). Although daily sleep duration was longer in the shift-work group, the PSQI total score was also significantly higher, indicating poorer sleep quality among shift workers (*p* < 0.001). In addition, DASS total, anxiety, and stress scores were significantly higher in the shift-work group than in the regular daytime schedule group. In contrast, the difference in depression scores did not reach statistical significance (Table 2).

Significant positive correlations were found among depression, anxiety, stress, and PSQI scores (*p* < 0.001). Strong correlations were observed among the DASS-21 subscales (r = 0.817–0.823), while the PSQI score showed moderate positive correlations with depression, anxiety, and stress scores (r = 0.500–0.570). These findings indicate that poorer sleep quality was associated with higher levels of psychological symptoms (Table 3).

When DASS total scores were compared according to sociodemographic, clinical, and work-related characteristics, no significant differences were observed in terms of age, sex, educational level, BMI, income status, occupation, years of professional experience, tobacco use, caffeine consumption, or mode of transportation to work (*p* > 0.05). In contrast, DASS total scores were significantly higher among unmarried participants, those without children, participants with chronic disease, those reporting negative mood status, and shift workers (*p* < 0.05) (Table 4).

DASS total scores were significantly higher among participants reporting sleepiness after work and among those reporting more frequent near-accident experiences (*p* < 0.001). In addition, DASS total scores were significantly higher among participants sleeping less than 6 h per day, those with higher PSQI scores, and those reporting job dissatisfaction (*p* < 0.001). Regarding work unit, participants working in administrative units had lower DASS scores than those working in other units (Table 4).

PSQI scores were compared according to sociodemographic, clinical, and work-related characteristics. No significant differences were found in PSQI scores according to age group, educational level, income status, years of professional experience, tobacco use, caffeine consumption, or mode of transportation to work (*p* > 0.05). However, PSQI scores were significantly higher among females, unmarried participants, those without children, participants with BMI < 25, nurses, participants with chronic disease, those reporting negative mood status, and shift workers (*p* < 0.05) (Table 5).

Regarding work unit, PSQI scores were higher among participants working in high-intensity clinical units and outpatient clinics, whereas those working in administrative units had lower PSQI scores (*p* = 0.002). PSQI scores were also significantly higher among participants reporting sleepiness after work, those with a higher frequency of near-accident experience, those reporting job dissatisfaction, and those sleeping less than 6 h per day (*p* < 0.001). These findings suggest that shift work and work-related sleepiness were associated with poorer sleep quality (Table 5).

In comparisons by job satisfaction status, significant differences were observed between groups in age, marital status, having children, BMI, work unit, work schedule, mood status, sleepiness after work, and daily sleep duration (*p* < 0.05). The proportion of participants reporting job satisfaction was higher among those aged 40 years or older, married participants, those with children, those working on a regular daytime schedule, those working in administrative units, those reporting positive mood status, those without sleepiness after work, and those reporting longer daily sleep duration (Table 6).

Participants with job dissatisfaction had significantly higher PSQI scores, resignation intention scores, DASS total scores, and depression, anxiety, and stress subscale scores (*p* < 0.001). No significant differences were found in terms of sex, educational level, income status, occupation, years of professional experience, chronic disease, perceived health status, tobacco or alcohol use, caffeine consumption, mode of transportation to work, near-accident experience, or type of near-accident (Table 6).

Multiple linear regression analysis was performed to identify factors associated with the PSQI total score. After adjustment for the other variables included in the model, the DASS-21 anxiety subscale score (B = 0.206, 95% CI: 0.074–0.338, standardized β = 0.227, *p* = 0.002), shift work (B = 2.295, 95% CI: 1.663–2.927, standardized β = 0.324, *p* < 0.001), and daily sleep duration (B = −1.269, 95% CI: −1.454 to −1.084, standardized β = −0.529, *p* < 0.001) were significantly associated with the PSQI total score. Higher anxiety scores and shift work were associated with higher PSQI scores, whereas longer daily sleep duration was associated with lower PSQI scores. The model explained 67.4% of the variance in PSQI total scores (R^2^ = 0.674; F = 31.337; *p* < 0.001) (Table 7).

Multiple linear regression analysis was performed to identify factors associated with the DASS total score. After adjustment for the other variables included in the model, reporting negative mood status (B = 4.146, 95% CI: 1.439–6.852, standardized β = 0.152, *p* = 0.003), sleepiness after work (B = 3.313, 95% CI: 0.779–5.847, standardized β = 0.139, *p* = 0.011), job dissatisfaction (B = 5.177, 95% CI: 2.690–7.663, standardized β = 0.219, *p* < 0.001), and the PSQI total score (B = 1.248, 95% CI: 0.878–1.618, standardized β = 0.376, *p* < 0.001) were significantly associated with higher DASS total scores. Marital status and the other variables included in the model were not statistically significant after adjustment. The model explained 46.5% of the variance in DASS total scores (R^2^ = 0.465; F = 14.898; *p* < 0.001) (Table 8).

Multivariable logistic regression analysis was performed to identify factors associated with job dissatisfaction. In the adjusted model, the DASS total score was the only variable significantly associated with job dissatisfaction (OR = 1.07, 95% CI: 1.03–1.11, *p* < 0.001). Each one-point increase in the DASS total score was associated with 7% higher odds of job dissatisfaction. None of the other variables included in the model was statistically significant (Table 9).

**Figure 1:**
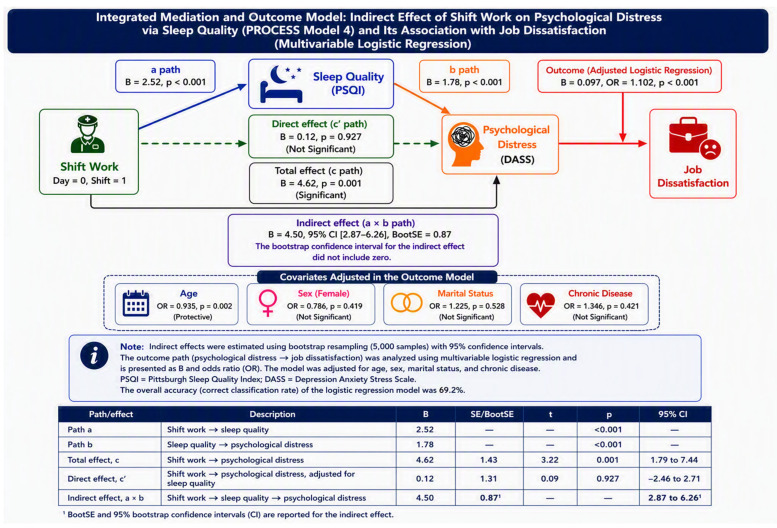
Integrated mediation and outcome model of shift work, sleep quality, psychological distress, and job dissatisfaction.

Mediation analysis was conducted using PROCESS Model 4 to examine whether the data were consistent with an indirect association between shift work and psychological distress through sleep quality. Shift work was associated with poorer sleep quality (path a: B = 2.52, *p* < 0.001), and poorer sleep quality was positively associated with psychological distress (path b: B = 1.78, *p* < 0.001). The total-effect coefficient was statistically significant (path c: B = 4.62, *p* = 0.001); however, after sleep quality was included in the model, the direct-effect coefficient was no longer statistically significant (path c′: B = 0.12, *p* = 0.927) (Figure 1).

The estimated indirect effect was 4.50 (BootSE = 0.87, 95% CI: 2.87–6.26). Because the bootstrap confidence interval did not include zero, the findings were consistent with a statistically significant indirect association between shift work and psychological distress through poorer sleep quality (Figure 1).

In the second stage of the analysis, multivariable logistic regression was used to evaluate the association between psychological distress and job dissatisfaction. After adjustment for the covariates included in the model, the DASS total score was associated with job dissatisfaction (B = 0.097, OR = 1.102, *p* < 0.001). Each one-point increase in the DASS total score was associated with approximately 10.2% higher odds of job dissatisfaction. Age was inversely associated with job dissatisfaction (OR = 0.935, *p* = 0.002), whereas sex, marital status, and presence of chronic disease were not significantly associated with the outcome (*p* > 0.05) (Figure 1).

## 4. Discussion

In this study, we examined the relationships between shift work, sleep quality, psychological distress, and job dissatisfaction among healthcare workers. Shift work was associated with poorer sleep quality, higher psychological distress, and greater job dissatisfaction. In addition, the mediation analysis showed a statistically significant indirect association between shift work and psychological distress through poorer sleep quality, whereas the direct association was not statistically significant after sleep quality was included in the model.

### 4.1. Shift Work and Sleep Quality

In the present study, shift workers had significantly higher PSQI scores than regular daytime workers, and shift work remained independently associated with poorer sleep quality in the multivariable analysis. An important contextual feature is that a substantial proportion of shift workers performed 24-h duties rather than conventional 8- or 12-h shifts. Such extended duties may entail different levels of fatigue accumulation and recovery needs. The magnitude of the observed association should therefore be interpreted within this local scheduling context and compared cautiously with findings from studies examining conventional shift systems.

Although shift workers reported longer daily sleep duration, their higher PSQI scores suggest that sleep duration alone did not adequately reflect overall sleep quality in this sample. Sleep timing, continuity, and restorative value may also be important. Circadian misalignment associated with night and irregular work may disrupt melatonin secretion, cortisol rhythms, body-temperature regulation, and the sleep–wake cycle, thereby impairing sleep initiation, maintenance, and restorative sleep [40]. The literature indicates that healthcare workers on night shifts experience greater difficulty falling asleep, maintaining sleep, and obtaining adequate sleep duration [13,41]. Batat et al. reported impaired sleep quality and altered sleep patterns among healthcare workers working night shifts [6]. Karhula et al. found that healthcare workers working night shifts were more prone to impairments in both sleep quality and sleep quantity due to difficulty falling asleep [42]. Lauren et al. reported that, compared with daytime workers, night shift workers experienced poorer sleep quality, felt less rested, and had shorter sleep episodes with substantially delayed timing [43]. Impaired sleep quality may be related not only to shift hours but also to job stress and working conditions. Lin et al. reported that job stress was associated with sleep quality among nurses, and that poor sleep quality was associated with poorer perceived health status [44]. Similarly, Choi and Kim found that job stress increased sleep disturbance among shift-working nurses [45]. However, some studies have reported no significant association between shift work and sleep quality [46,47]. These inconsistent findings may be explained by differences in sample characteristics, shift type, frequency of night shifts, work unit, cultural factors, and workload. Therefore, when evaluating the relationship between shift work and sleep quality, not only the presence of shift work but also the nature of the shift schedule and the broader working conditions should be considered.

### 4.2. Shift Work and Psychological Distress

In this study, shift workers had significantly higher DASS total scores, anxiety scores, and stress scores than those working on a regular daytime schedule. Depression scores were also higher among shift workers, although this difference did not reach statistical significance.

The higher anxiety and stress scores among shift workers may reflect the combined biological, psychological, and social consequences of shift work. Shift work may increase psychological burden through circadian disruption, reduced sleep quality, insufficient recovery after work, limited social life, and disruption of family roles. For healthcare workers, shift work does not simply mean a change in working hours; it is also a structural aspect of work organisation accompanied by intensive clinical responsibilities, patient care burden, the need for rapid decision-making, and emotional labour [13,31,43].

The literature suggests that shift work may have multidimensional adverse effects on mental health. Shift work has been associated with sleep disruption, depressive mood, anxiety, reduced cognitive functioning, lower quality of life, and other psychosocial problems. Sleep disturbances related to shift work are often considered central to these effects [31]. In addition, shift work has been reported to be significantly associated with perceived stress, which may be explained by reduced opportunities for socialisation during rotating shifts, heavy workload, high responsibility, burnout, insufficient rewards, and poor sleep quality [47]. The higher anxiety and stress scores observed among shift workers in the present study support the view that shift work is not only a work pattern that disrupts biological rhythms but also a multidimensional source of stress affecting social life, recovery, workload, and psychological resilience.

Job stress may be an important factor that strengthens the relationship between shift work and sleep and overall health. Lin et al. reported that as job stress increased among nurses, sleep quality scores also increased, indicating poorer sleep quality; poorer sleep quality was also associated with more negative perceived health status. Persistent high job stress may impair sleep quality and adversely affect physical and psychological health, as well as absenteeism and work performance [44]. This finding is consistent with the more unfavourable indicators of sleep quality and psychological distress observed among shift workers in the present study.

Our findings suggest that shift work is particularly associated with higher anxiety and stress levels among healthcare workers. However, the loss of independent significance of shift work in the multivariable analysis suggests that this relationship may not be direct, but may operate through intermediate factors such as sleep quality, job stress, job dissatisfaction, disruption of social life, and insufficient recovery after work. Therefore, when evaluating the relationship between shift work and psychological distress, not only the presence of shift work but also shift intensity, sleep quality, workload, social support, job satisfaction, and recovery processes should be considered together. Accordingly, interventions should not focus solely on reducing the number of shifts. Still, they should also include institutional arrangements that improve sleep quality, reduce job stress, balance workloads, strengthen social support mechanisms, and support healthier shift scheduling.

### 4.3. Relationship Between Sleep Quality and Psychological Symptoms

In this study, significant positive correlations between PSQI scores and DASS subscale scores indicate that impaired sleep quality is closely related to psychological symptom burden. The moderate correlations between PSQI scores and depression, anxiety, and stress scores suggest that poor sleep quality may affect not only physical rest but also psychological well-being among healthcare workers. Similarly, a study using both DASS-21 and PSQI reported that sleep disturbances were associated with depression, anxiety, and stress among nurses [48]. Another study conducted among clinical nurses also demonstrated positive associations between PSQI scores and anxiety and depression scores [49].

The strong correlations among the DASS subscales suggest that depressive symptoms, anxiety symptoms, and stress symptoms among healthcare workers may not be completely independent processes, but rather different manifestations of a common pattern of psychological distress. The relationship between sleep disturbances and psychological symptoms may be bidirectional. Poor sleep quality may increase the stress response, impair emotional regulation, and weaken cognitive control mechanisms. Conversely, depressive, anxiety, and stress symptoms may contribute to sleep problems such as difficulty falling asleep, frequent awakenings, early morning awakening, and waking up feeling unrefreshed. Indeed, among nurses working night shifts, higher PSQI and depression scores have been reported, and poor sleep quality has been identified as a potential independent factor associated with depressive symptoms [50]. Although the cross-sectional design of the present study does not allow determination of the direction of this relationship, the mediation model suggests that sleep quality may be an important intermediate mechanism linking shift work to psychological distress.

### 4.4. Psychological Distress and Job Dissatisfaction

One of the important findings of this study was that psychological distress was independently associated with job dissatisfaction. The significantly higher DASS total score and depression, anxiety, and stress subscale scores among workers reporting job dissatisfaction indicate that job satisfaction cannot be explained only by external working conditions such as salary, work unit, or shift schedule. The fact that the DASS total score remained significantly associated with job dissatisfaction in the multivariable logistic regression analysis suggests that psychological burden may play a central role in job satisfaction. This finding is consistent with studies emphasising the multidimensional nature of job satisfaction. Batat et al., in their study examining relationships among night shifts, sleep patterns, psychological well-being, and mental health, reported that job satisfaction, social life, anxiety, and depression were influenced not only by shift schedules but also by many individual and environmental factors [6]. Rodwell and Fernando also emphasised that job satisfaction was related not only to shift type but also to job characteristics, lifestyle factors, and the broader context in which employees work [51]. In this regard, the independent association of the DASS total score with job dissatisfaction in the present study supports the close relationship between job dissatisfaction and psychological distress.

In the present study, participants with job dissatisfaction had markedly higher scores on resignation intention. Similarly, Batat et al. reported that lower job satisfaction was associated with higher levels of anxiety and depression, and that the negative impact of working hours on social life may increase psychological symptom burden [6]. These findings suggest that job dissatisfaction is not merely an indicator of individual discontent but also an important marker of employee commitment and intention to leave. Our study indicates that psychological distress is an important factor independently associated with job dissatisfaction among healthcare workers. Therefore, creating supportive work environments that reduce psychological distress, balance workloads, increase managerial and team support, and develop practices that support sleep and rest patterns are critical for both employee well-being and workforce continuity.

### 4.5. Job Satisfaction, Resignation Intention, and Workforce Sustainability

It is noteworthy that nearly half of the participants in this study reported dissatisfaction with their jobs. The finding that job dissatisfaction was more prominent among shift workers, those working in clinical units, those reporting sleepiness after work, short sleepers, and those with higher DASS and PSQI scores suggests that job satisfaction is a multidimensional outcome that a single factor cannot explain. This finding is consistent with studies reporting lower job satisfaction among healthcare workers working night shifts compared with daytime workers. Batat et al. reported that night shift workers had higher job dissatisfaction and were more likely to seek other job opportunities [6]. Similarly, Qanash et al. found that job satisfaction was lower among healthcare workers working night shifts than among those working daytime shifts [13]. The relationship between night shift work and job dissatisfaction may be explained by sleep deprivation, disruption of social life, psychological distress, and reduced quality of life. A study among healthcare workers on rotating night shifts found that more hours on night shifts increased the likelihood of low job satisfaction [52]. Sánchez-Sellero et al. found that job satisfaction was lowest among rotating-shift workers and that fixed night-shift workers had lower job satisfaction than daytime workers [53]. These findings are consistent with the more prominent job dissatisfaction observed among shift workers in the present study. However, some studies have reported no significant difference in job satisfaction between nurses working night and day shifts [54]. These differences may be related to variations in sample characteristics, shift type, frequency of night shifts, work unit, workload, institutional support, and social life conditions.

In this study, the most commonly reported reasons for job dissatisfaction were emotional burden and interpersonal interactions. Healthcare workers are exposed not only to physical workload but also to intense emotional labour arising from interactions with patients, patients’ relatives, and team members. When combined with shift work, sleep disturbance, and psychological distress, this emotional labour may further reduce job satisfaction.

The significantly higher resignation intention scores among participants with job dissatisfaction suggest that this finding is not only an indicator of individual dissatisfaction but also an important warning sign for institutional workforce loss. This finding is supported by previous studies [55,56,57,58]. Low job satisfaction may lead to reduced employee commitment, increased job-search activity, and greater intention to leave. In healthcare systems, increased staff turnover may result in the loss of experienced personnel, increased adaptation burden for new staff, disruption of continuity of care, and decreased service quality.

### 4.6. Sociodemographic and Occupational Differences

In this study, shift work was more common among younger participants, males, unmarried participants, those without children, nurses, those working in clinical units, and those with shorter professional experience. This distribution suggests that the burden of shift work is not evenly distributed across all employee groups in healthcare institutions but is concentrated among specific occupational and demographic groups. The greater involvement of younger and less experienced workers in shift schedules may be related to career stage, seniority, and internal task allocation.

The higher prevalence of shift work among nurses and employees working in clinical units is expected. Because healthcare services must be provided continuously, nurses and clinical staff directly involved in patient care are more likely to work night and rotating shifts. The literature also indicates that shift work may lead to psychological, social, physical, and sleep-related outcomes among healthcare workers, and that these effects may vary according to speciality area, workload, and frequency of night shifts [13,59,60].

The higher proportion of male employees among shift workers in this study may be attributable to the sample’s occupational distribution and institutional shift planning. However, the literature suggests that the effects of shift work may differ by sex, and some studies have reported that impaired sleep quality and work–family conflict may be more pronounced among female workers [60,61]. Therefore, shift planning should consider not only sex but also family responsibilities, social support, occupational role, and clinical workload.

### 4.7. Practical and Managerial Implications

The findings of this study offer several practical and managerial implications for healthcare institutions. Shift work should be viewed not solely as an operational arrangement required to maintain continuity of care, but also as an organisational factor that may be associated with sleep quality, psychological distress, job satisfaction, patient safety, and workforce sustainability. Accordingly, hospital administrators and institutional decision-makers should regularly assess the potential effects of shift arrangements on employees’ recovery time, sleep health, and psychological well-being.

A primary goal of shift scheduling should be to ensure adequate recovery time between shifts. Quick returns, particularly intershift intervals shorter than 11 h, have been associated with insufficient recovery and adverse outcomes related to sleep, health, and work performance [62]. Hospital-based intervention studies have reported that reducing quick returns may produce small but meaningful improvements in insomnia and daytime sleepiness [63]. Evidence also suggests that longer recovery periods may be required after consecutive long shifts; at least three rest days may be needed for full recovery after two consecutive 12-h day or night shifts [64,65]. Healthcare institutions should therefore avoid excessively short intershift intervals and abrupt shift rotations, limit consecutive night shifts whenever feasible, and provide adequate blocks of time off, particularly following 24-h duties or periods of intensive clinical workload [66]. In the present study, the fact that a substantial proportion of shift workers performed 24-h duties indicates that local scheduling practices should be carefully reviewed in relation to both staffing requirements and employees’ recovery needs.

Organisational scheduling arrangements should be predictable, and participatory scheduling—a model in which employees actively contribute to schedule planning within established rules and unit requirements—should be supported rather than having shifts assigned solely by managers [67]. Unpredictable schedules, last-minute changes, understaffing, and overtime may adversely affect recovery and work–life balance [66,68]. Healthcare institutions could therefore increase schedule predictability, use digital tools to monitor compliance with statutory rest periods and breaks, and treat recovery as a measurable occupational health indicator. Participatory scheduling and greater employee control over shift arrangements may strengthen autonomy, job satisfaction, and perceived organisational support [68,69].

Sleep-health initiatives should be designed as multicomponent programs rather than single educational sessions. Programs for healthcare workers on night shifts may be more meaningful when they combine designated facilities for short naps or rest, healthy food options, sleep-hygiene education, fatigue-management training, e-learning, and sleep coaching [70]. Nevertheless, given the cross-sectional design of the present study, these approaches should be considered potential strategies rather than interventions whose causal effectiveness is established by our findings.

Regarding psychological well-being, the most useful approach may not be to focus solely on strengthening individual resilience, but to reinforce organisational support mechanisms. Evidence suggests that work and task redesign, flexible scheduling, improvements in the physical work environment, supportive leadership, peer support, accessible mental health counselling, and greater employee autonomy may support psychological well-being among shift workers [66,71]. When designing interventions to reduce psychological distress and job dissatisfaction in healthcare institutions, workload distribution, emotional demands, interpersonal conflict, managerial support, team communication, and opportunities for recovery should therefore be considered together.

Finally, the success of these interventions depends not only on their content but also on the context in which they are implemented. In a multicomponent night-work intervention, the most important facilitators were identified at the organisational level; a positive institutional culture, clear implementation procedures, manageable workloads, control over scheduling, opportunities for breaks, and local implementation champions or night-shift representatives may facilitate intervention adoption and sustainability [70,72]. Healthcare institutions should therefore avoid implementing standardised shift-work interventions without considering local workloads, staffing structures, unit-specific requirements, and implementation capacity. Future prospective and intervention studies are needed to determine whether organisational and sleep-related interventions improve sleep health, reduce psychological distress, and increase job satisfaction among healthcare workers.

### 4.8. Strengths of the Study

This study has several strengths. First, it evaluated the relationships among shift work, sleep quality, psychological distress, and job dissatisfaction not only through pairwise comparisons but also within an integrated analytical model. This approach contributes to a better understanding of the possible mechanisms linking shift work with employee well-being. Second, valid and reliable measurement tools, such as the DASS-21 and the Pittsburgh Sleep Quality Index, were used. In addition, mediation analysis was used to evaluate the possible intermediate role of sleep quality in the association between shift work and psychological distress, and job dissatisfaction was examined using a multivariable logistic regression model. In this respect, the study addresses the effects of shift work among healthcare workers from the perspectives of both individual well-being and workforce sustainability.

### 4.9. Limitations

This study has several limitations. First, its cross-sectional design precludes conclusions about temporality or causality. Accordingly, the mediation findings should be interpreted as statistical associations consistent with the proposed theoretical model rather than as evidence of a causal pathway. Longitudinal studies are needed to clarify the temporal relationships among shift work, sleep quality, psychological distress, and job dissatisfaction.

Second, this was a single-centre study with voluntary participation, which may limit the generalizability of the findings to other hospitals, regions, organisational structures, and healthcare systems. Voluntary participation may also have introduced selection bias: workers concerned about sleep, psychological symptoms, or job dissatisfaction may have been more likely to participate, whereas those with heavier workloads, limited time, or more severe symptoms may have been less likely to respond. Furthermore, a substantial proportion of shift workers performed 24-h duties, and shift schedules could not be examined in detail by subgroup. The findings should therefore be interpreted in the context of local scheduling practices and compared cautiously with studies of conventional 8- or 12-h shift systems.

Third, the data were collected through self-report measures and may therefore be affected by recall bias, reporting bias, social desirability bias, and common-method variance. Although validated instruments were used to assess sleep quality and psychological distress, objective measures of sleep or circadian disruption, such as actigraphy, polysomnography, or biomarker-based assessments, were not available. In addition, job satisfaction was assessed using a single self-reported item rather than a validated multidimensional scale. Consequently, the measure may not fully capture dimensions such as autonomy, workload, salary, professional recognition, interpersonal relationships, leadership support, and career development.

Finally, excluding workers with previously diagnosed sleep disorders, anxiety, or depression may have produced a healthy-worker selection effect and resulted in underrepresentation of employees with more severe sleep or mental health problems. Residual confounding also cannot be ruled out, as potentially relevant factors—including workload, patient volume, night-shift frequency, rest intervals, social support, burnout, organisational justice, work–life balance, and managerial support—were not fully captured. Future multicenter longitudinal and intervention studies should incorporate detailed shift characteristics, institutional and psychosocial factors, objective sleep measures, and validated multidimensional job-satisfaction instruments.

## 5. Conclusions

This study found that shift work was associated with poorer sleep quality and greater psychological distress among healthcare workers, while psychological distress was independently associated with job dissatisfaction. The mediation model identified a significant indirect association between shift work and psychological distress through sleep quality; however, the cross-sectional design precludes temporal or causal inferences. The findings should also be interpreted in the context of the local scheduling practice, in which many shift workers performed 24-h duties. These results highlight sleep health and psychological well-being as important organisational concerns. Strategies promoting adequate recovery time, safer and more predictable shift schedules, and psychosocial support should be considered, while their effectiveness should be evaluated in prospective and interventional studies.

## Figures and Tables

**Table 1:** Sociodemographic, occupational, lifestyle, and health-related characteristics of the participants.

Variables		Number/%
Age		33.46 ± 7.74 32 (20–59)
Age interval	20–29	108 (39.1)
	30–39	108 (39.1)
	40–49	50 (18.1)
	50–59	10 (3.6)
Sex	Male	90 (32.6)
	Female	186 (67.4)
Educational level	Primary school	1 (0.4)
	Secondary school	1 (0.4)
	High school	31 (11.2)
	Associate degree	1 (0.4)
	Bachelor’s degree	234 (84.7)
	Master’s/Doctoral degree	8 (2.9)
Marital status	Single	102 (37.0)
	Married	164 (59.4)
	Divorced-Widowed	10 (3.6)
Height		167.63 ± 8.26 167 (146–195)
Weight		70.20 ± 12.60 70 (48–105)
BMI	Below 18.5 Underweight	4 (1.4)
	18.5–24.9 Normal	150 (54.3)
	25–29.9 Overweight	98 (35.5)
	30–39.9 Obese	24 (8.7)
Number of children	None	130 (47.1)
	1–2	105 (38.0)
	3 and above	41 (14.9)
Income status	2–3 times the minimum wage	231 (83.7)
	More than 3 times the minimum wage	45 (16.3)
Occupation	Nurse	202 (73.2)
	Medical secretary/Data entry personnel	74 (26.8)
Work unit	Emergency department	107 (38.8)
	Outpatient clinic	73 (26.4)
	Inpatient ward	43 (15.6)
	Administrative unit	31 (11.2)
	Surgical unit/Operating room	22 (8.0)
Years of professional experience	Below 2 years	68 (24.6)
	2–5 years	39 (14.1)
	5–8 years	36 (13.0)
	Above 8 years	133 (48.2)
Monthly overtime status	No	235 (85.1)
	Yes	41 (14.9)
Most common work schedule	Regular daytime schedule	**135 (48.9)**
	Shift work schedule	**141 (51.1)**
	08:00–08:00, 24-h duty	110 (78.0)
	08:00–20:00, 12-h shift	11 (7.8)
	20:00–08:00, 12-h shift	5 (3.5)
	16:00–08:00, 16-h shift	9 (6.4)
	Rotating schedule outside regular working hours	6 (4.3)
Presence of chronic disease	Yes	56 (20.3)
	No	220 (79.7)
Mood status in the previous month	Negative mood status	208(75.4)
	Depressive	75 (27.2)
	Anxious	133 (48.2)
	Positive mood status	68 (24.6)
	Excited	11 (4.0)
	Happy	57 (20.7)
How healthy do you feel?		6.16 ± 1.97 6 (1–10)
Tobacco product use	Never used	178 (64.5)
	Current user	80 (29.0)
	Former user	18 (6.5)
Pack-years among tobacco product users (*n* = 80)		11.34 ± 7.10 11 (0.42–32.00)
Type of tobacco product (*n* = 80)	Packaged cigarettes	64 (80.0)
	Hand-rolled cigarettes	14 (17.5)
	Waterpipe	2 (2.5)
Alcohol use	No	257 (93.1)
	Yes	19 (6.9)
Number of caffeinated beverages per day	1–2	105 (38.0)
	3–4	88 (31.9)
	5 and above	83 (30.1)
Mode of transportation to work	Private vehicle	144 (52.2)
	Public transportation	86 (31.1)
	Walking	46 (16.7)
Sleepiness after work	No	117 (42.4)
	Yes	159 (57.6)
Frequency of near-accident experience	None	112 (40.6)
	1–2	120 (43.5)
	3–4	33 (12.0)
	5–6	5 (1.8)
	7 and above	6 (2.2)
Type of near-accident (*n* = 164)	Home	80 (48.8)
	Traffic	77 (46.9)
	Work	7 (4.3)
Daily sleep duration	Below 6 h	78 (28.3)
	6–8 h	153 (55.4)
	Above 8 h	45 (16.3)
Job satisfaction	Yes	149 (54.0)
	No	127 (46.0)
Reason for job dissatisfaction (*n* = 127)	Dealing with people/hiding emotions while working	68 (53.5)
	Financial difficulties	31 (24.4)
	Work schedule/colleagues/time-related problems	23 (18.1)
	Health problem	5 (4.0)
Resignation intention		4.46 ± 2.93
DASS total score		22.77 ± 11.76
Depression		7.57 ± 4.47
Anxiety		6.95 ± 3.89
Stress		8.25 ± 4.16
Pittsburgh Sleep Quality Index (PSQI)		9.08 ± 3.54

Bold values indicate significance.

**Table 2:** Comparison of sociodemographic, occupational, health-related, and psychosocial characteristics of participants according to work schedule.

Variables		Regular (*n* = 135)	Shift (*n* = 141)	*p* Values
Age	20–29 ^a^	33 (30.6)	75 (69.4)	**X^2^ = 24.185** ***p* < 0.001**
	30–39 ^b^	64 (59.3)	44 (40.7)	
	40 and above ^b^	38 (63.3)	22 (36.7)	
Sex	Male	32(35.6)	58 (64.4)	**X^2^ = 9.536** ***p* = 0.002**
	Female	103 (55.4)	83 (44.6)	
Education level	Associate degree and below	17 (50.0)	17 (50.0)	X^2^ = 0.018 *p* = 0.892
	Bachelor’s degree and above	118 (48.8)	124 (51.2)	
Marital status	Married	97 (59.1)	67 (40.9)	**X^2^ = 16.937** ***p* < 0.001**
	Not married	38 (33.9)	74 (66.1)	
Presence of children	None	44 (33.8)	86 (66.2)	**X^2^ = 22.326** ***p* < 0.001**
	Yes	91 (62.3)	55 (37.7)	
BMI	Below 25	64 (41.6)	90 (58.4)	**X^2^ = 7.541** ***p* = 0.006**
	25 and above	71 (58.2)	51 (41.8)	
Income status	2–3 times the minimum wage	110 (47.6)	121 (52.4)	X^2^ = 0.949 *p* = 0.330
	More than 3 times the minimum wage	25 (55.6)	20 (44.4)	
Occupation	Medical secretary/Data entry personnel	49 (66.2)	25 (33.8)	**X^2^ = 12.115** ***p* = 0.001**
	Nurse	86 (42.6)	116 (57.4)	
Work Unit	Emergency/Inpatient/Surgical/Operating ^a^	57 (33.1)	115 (66.9)	**X^2^ = 56.495** ***p* < 0.001**
	Outpatient clinic ^b^	47 (64.4)	26 (35.6)	
	Administrative unit ^c^	31 (100.0)	0 (0.0)	
Years of professional experience	Below 2 years ^a^	23 (33.8)	45 (66.2)	**X^2^ = 23.403** ***p* < 0.001**
	2–5 years ^a^	13 (33.3)	26 (66.7)	
	5–8 years ^a^	14 (38.9)	22 (61.1)	
	Above 8 years ^b^	85 (63.9)	48 (36.1)	
Presence of chronic disease	No	100 (45.5)	120 (54.5)	**X^2^ = 5.190** ***p* = 0.023**
	Yes	35 (62.5)	21 (37.5)	
Mood status	Negative	100 (48.1)	108 (51.9)	X^2^ = 0.236 *p* = 0.627
	Positive	35 (51.5)	33 (48.5)	
Perception of health status		5.96 ± 1.85	6.35 ± 2.07	t = −1.662 *p* = 0.098
Tobacco product use	Others	92 (46.9)	104 (53.1)	X^2^ = 1.055 *p* = 0.304
	Current user	43 (53.8)	37 (46.2)	
Alcohol use	No	127 (49.4)	130 (50.6)	X^2^ = 0.378 *p* = 0.538
	Yes	8 (42.1)	11 (57.9)	
Caffeinated beverages per day	1–2	47 (44.8)	58 (55.2)	X^2^ = 1.169 *p* = 0.280
	3 and above	88 (51.5)	83 (48.5)	
Mode of transportation to work	Walking	19 (41.3)	27 (58.7)	X^2^ = 1.419 *p* = 0.492
	Public transportation	42 (48.8)	44 (51.2)	
	Private vehicle	74 (51.4)	70 (48.6)	
Sleepiness after work	No	77 (65.8)	40 (34.2)	**X^2^ = 23.210** ***p* < 0.001**
	Yes	58 (36.5)	101 (63.5)	
Frequency of near-accident experience	None	54 (48.2)	58 (51.8)	X^2^ = 1.768 *p* = 0.413
	1–2	63 (52.5)	57 (47.5)	
	3 and above	18 (40.9)	26 (59.1)	
Type of near-accident (*n* = 164)	Home	39 (48.8)	41 (51.2)	X^2^ = 0.181 *p* = 0.913
	Traffic	39 (50.6)	38 (49.4)	
	Work	3 (42.9)	4 (57.1)	
Daily sleep duration	Below 6 h ^a^	46 (59.0)	32 (41.0)	**X^2^ = 10.416** ***p* = 0.005**
	6–8 h ^a^	76 (49.7)	77 (50.3)	
	Above 8 h ^b^	13 (28.9)	32 (71.1)	
Job satisfaction	Yes	93 (62.4)	56 (37.6)	**X^2^ = 23.628** ***p* < 0.001**
	No	42 (33.1)	85 (66.9)	
Reason for the job dissatisfaction (*n* = 127)	Dealing with people/hiding emotions while working ^a^	24 (35.3)	44 (64.7)	**X^2^ = 9.528** ***p* = 0.023**
	Financial difficulties	9 (39.1)	14 (60.9)	
	Work schedule/colleagues/time-related problems ^a^	5 (16.1)	26 (83.9)	
	Health problem ^b^	4 (80.0)	1 (20.0)	
Resignation intention		3.60 ± 2.62	5.29 ± 2.98	**t = −5.011** ***p* < 0.001**
DASS total score		20.28 ± 13.43	25.16 ± 9.35	**t = −3.489** ***p* < 0.001**
Depression		7.11 ± 5.06	8.01 ± 3.80	t = −1.643 *p* = 0.102
Anxiety		6.01 ± 4.48	7.85 ± 2.97	**t = −3.991** ***p* < 0.001**
Stress		7.14 ± 4.62	9.30 ± 3.37	**t = −4.414** ***p* < 0.001**
Pittsburgh Sleep Quality Index (PSQI)		7.90 ± 3.48	10.21 ± 3.22	**t = −5.733** ***p* < 0.001**

χ^2^: Chi-square test; t: independent samples *t*-test; ^a,b,c^ different superscript letters indicate statistically significant differences between groups. Bold values indicate significance.

**Table 3:** Correlations between DASS-21 subscale scores and the PSQI score.

Variable	1	2	3	4
1. Depression	1			
2. Anxiety	0.823 *	1		
3. Stress	0.817 *	0.823 *	1	
4. PSQI score	0.500 *	0.570 *	0.555 *	1

* Pearson correlation coefficients; all correlations were statistically significant at *p* < 0.001.

**Table 4:** Comparison of DASS-21 total scores according to sociodemographic, occupational, and health-related characteristics.

Variables		Mean ± SD	*p*, F, t Values
Age groups	20–29	24.14 ± 11.48	F = 1.243 *p* = 0.290
	30–39	22.11 ± 11.11	
	40 and above	21.52 ± 13.27	
Sex	Male	21.26 ± 11.52	t = −1.496 *p* = 0.136
	Female	23.51 ± 11.85	
Education level	Associate degree and below	23.20 ± 14.82	t = 0.186 *p* = 0.854
	Bachelor’s degree and above	22.71 ± 11.30	
Marital status	Not married	26.03 ± 11.91	**t = 3.890** ***p* < 0.001**
	Married	20.56 ± 11.17	
Presence of children	None	24.86 ± 12.14	**t = 2.814** ***p* = 0.005**
	Yes	20.91 ± 11.13	
BMI	Below 25	23.15 ± 11.15	F = 0.289 *p* = 0.749
	25–29.9	22.54 ± 12.56	
	30–39.9	21.29 ± 12.58	
Income status	2–3 times the minimum wage	22.97 ± 11.94	t = 0.649 *p* = 0.517
	More than 3 times the minimum wage	21.73 ± 10.86	
Occupation	Medical secretary/Data entry personnel	21.72 ± 13.72	t = 0.806 *p* = 0.422
	Nurse	23.15 ± 10.97	
Work unit	Emergency/Inpatient/Surgical/Operating ^a^	23.25 ± 10.12	**F = 3.255** ***p* = 0.040**
	Outpatient clinic ^a^	23.76 ± 14.86	
	Administrative unit ^b^	17.77 ± 11.15	
Years of professional experience	Below 2 years	25.08 ± 10.76	F = 1.763 *p* = 0.154
	2–5 years	20.82 ± 10.91	
	5–8 years	24.25 ± 9.71	
	Above 8 years	21.76 ± 12.83	
Work schedule	Regular	20.28 ± 13.43	**t = −3.489** ***p* < 0.001**
	Shift	25.16 ± 9.35	
Presence of chronic disease	No	21.94 ± 10.90	**t = −2.005** ***p* = 0.049**
	Yes	26.05 ± 14.32	
Mood status	Negative	25.01 ± 11.79	**t = 6.846** ***p* < 0.001**
	Positive	15.91 ± 8.65	
Tobacco product use	Other	22.13 ± 11.33	t = −1.423 *p* = 0.156
	Current user	24.35 ± 12.70	
Caffeinated beverages per day	1–2	21.46 ± 12.02	t = −1.451 *p* = 0.148
	3 and above	23.57 ± 11.56	
Mode of transportation to work	Walking	25.39 ± 13.77	F = 1.369 *p* = 0.256
	Public transportation	22.30 ± 11.66	
	Private vehicle	22.22 ± 11.08	
Sleepiness after work	No	17.58 ± 9.75	**t = 6.787** ***p* < 0.001**
	Yes	26.59 ± 11.68	
Frequency of near-accident experience	None ^a^	20.20 ± 10.19	**F = 3.255** ***p* < 0.001**
	1–2 ^a,b^	23.38 ± 11.79	
	3 and above ^b^	27.65 ± 13.75	
Type of near-accident (*n* = 164)	Home	23.60 ± 12.52	X^2^ = 3.471 *p* = 0.176
	Traffic	24.96 ± 12.60	
	Work	30.42 ± 9.23	
Daily sleep duration	Below 6 h ^a^	27.42 ± 14.08	**F = 9.002** ***p* < 0.001**
	6–8 h ^b^	20.84 ± 10.58	
	Above 8 h ^b^	21.28 ± 8.79	
Job satisfaction	Yes	17.91 ± 9.53	**t = −8.177** ***p* < 0.001**
	No	28.48 ± 11.60	
PSQI score 5 and above	Yes (*n* = 256)	23.88 ± 11.27	**U = 563.000** ***p* < 0.001**
	No (*n* = 20)	8.60 ± 8.33	
PSQI score 8 and above	Yes (*n* = 176)	26.36 ± 11.05	**t = 7.339** ***p* < 0.001**
	No (*n* = 100)	16.46 ± 10.27	

t: independent samples *t*-test; F: one-way ANOVA; U: Mann–Whitney U test; χ^2^: Kruskal–Wallis H test.; ^a,b^ different superscript letters indicate statistically significant differences between groups. Bold values indicate significance.

**Table 5:** Comparison of PSQI total scores according to sociodemographic, occupational, and health-related characteristics.

Variables		Mean ± SD	*p*, F, t Values
Age groups	20–29	9.65 ± 3.37	F = 2.713 *p* = 0.068
	30–39	8.89 ± 3.62	
	40 and above	8.40 ± 3.59	
Sex	Male	8.34 ± 3.15	**t = −2.576** ***p* = 0.011**
	Female	9.44 ± 3.67	
Education level	Associate degree and below	9.23 ± 4.79	t = 0.199 *p* = 0.843
	Bachelor’s degree and above	9.06 ± 3.34	
Marital status	Not married	9.72 ± 3.47	**t = −2.497** ***p* = 0.013**
	Married	8.65 ± 3.53	
Presence of children	None	9.59 ± 3.58	**t = −2.252** ***p* = 0.025**
	Yes	8.63 ± 3.45	
BMI	Below 25 ^a^	9.59 ± 3.53	**F = 3.626** ***p* = 0.028**
	25–29.9 ^b^	8.40 ± 3.20	
	30–39.9 ^a,b^	8.62 ± 4.48	
Income status	2–3 times the minimum wage	9.12 ± 3.53	t = 0.409 *p* = 0.683
	More than 3 times the minimum wage	8.88 ± 3.60	
Occupation	Medical secretary/Data entry personnel	8.36 ± 3.73	**t = 2.061** ***p* = 0.040**
	Nurse	9.35 ± 3.44	
Work unit	Emergency/Inpatient/Surgical/Operating ^a^	9.26 ± 3.28	**F = 6.244** ***p* = 0.002**
	Outpatient clinic ^a^	9.53 ± 3.87	
	Administrative unit ^b^	7.03 ± 3.57	
Years of professional experience	Below 2 years	9.51 ± 3.63	F = 1.186 *p* = 0.315
	2–5 years	9.12 ± 3.17	
	5–8 years	9.66 ± 3.02	
	Above 8 years	8.69 ± 3.71	
Work schedule	Regular	7.90 ± 3.48	**t = −5.733** ***p* < 0.001**
	Shift	10.21 ± 3.22	
Presence of chronic disease	No	8.84 ± 3.36	**t = 2.026** ***p* = 0.025**
	Yes	10.03 ± 4.05	
Mood status	Negative	9.64 ± 3.46	**t = −4.744** ***p* < 0.001**
	Positive	7.38 ± 3.23	
Tobacco product use	Other	8.96 ± 3.45	t = 0.900 *p* = 0.369
	Current user	9.38 ± 3.75	
Caffeinated beverages per day	1–2	8.60 ± 3.29	t = 0.900 *p* = 0.369
	3 and above	9.38 ± 3.61	
Mode of transportation to work	Walking	9.52 ± 3.76	F = 0.450 *p* = 0.638
	Public transportation	8.91 ± 3.56	
	Private vehicle	9.04 ± 3.46	
Sleepiness after work	No	7.72 ± 3.10	**t = 5.785** ***p* < 0.001**
	Yes	10.08 ± 3.52	
Frequency of near-accident experience	None ^a^	8.17 ± 3.06	**F = 6.868** ***p* = 0.001**
	1–2 ^b^	9.55 ± 3.67	
	3 and above ^b^	10.11 ± 3.84	
Type of near-accident (*n* = 164)	Home	9.71 ± 3.67	X^2^ = 2.272 *p* = 0.321
	Traffic	9.55 ± 3.86	
	Work	11.28 ± 2.21	
Daily sleep duration	Below 6 h ^a^	12.25 ± 3.26	**F = 70.400** ***p* < 0.001**
	6–8 h ^b^	8.18 ± 2.73	
	Above 8 h ^c^	6.64 ± 2.69	
Job satisfaction	Yes	7.85 ± 3.08	**t = −6.716** ***p* < 0.001**
	No	10.52 ± 3.52	

t: independent samples *t*-test; F: one-way ANOVA; χ^2^: Kruskal–Wallis test statistic.; ^a,b,c^ different superscript letters indicate statistically significant differences between groups. Bold values indicate significance.

**Table 6:** Comparison of sociodemographic, occupational, health-related, and psychosocial characteristics according to job satisfaction status.

Variables		Satisfied (*n* = 149)	Dissatisfied (*n* = 127)	*p*, X^2^ Values
Age groups	20–29 ^a^	51 (47.2)	57 (52.8)	**X^2^ = 11.721** ***p* = 0.003**
	30–39 ^a^	54 (50.0)	54 (50.0)	
	40 and above ^b^	44 (73.3)	16 (26.7)	
Sex	Male	48 (53.3)	42 (46.7)	X^2^ = 0.023 *p* = 0.880
	Female	101 (54.3)	85 (45.7)	
Education level	Associate degree and below	20 (58.8)	14 (41.2)	X^2^ = 0.365 *p* = 0.546
	Bachelor’s degree and above	129 (53.3)	113 (46.7)	
Marital status	Married	99 (60.4)	65 (39.6)	**X^2^ = 6.623** ***p* = 0.010**
	Not married	50 (44.6)	62 (55.4)	
Presence of children	None	60 (46.2)	70 (53.8)	**X^2^ = 6.068** ***p* = 0.014**
	Yes	89 (61.0)	57 (39.0)	
BMI	Below 25	71 (46.1)	83 (53.9)	**X^2^ = 8.712** ***p* = 0.003**
	25 and above	78 (63.9)	44 (36.1)	
Income status	2–3 times the minimum wage	123 (53.2)	108 (46.8)	X^2^ = 0.311 *p* = 0.577
	More than 3 times the minimum wage	26 (57.8)	19 (42.2)	
Occupation	Medical secretary/Data entry personnel	42 (56.8)	32 (43.2)	X^2^ = 0.313 *p* = 0.576
	Nurse	107 (53.0)	95 (47.0)	
Work unit	Emergency/Inpatient/Surgical/Operating ^a^	84 (48.8)	88 (51.2)	**X^2^ = 15.626** ***p* < 0.001**
	Outpatient clinic ^a^	38 (52.1)	35 (47.9)	
	Administrative unit ^b^	27 (87.1)	4 (12.9)	
Years of professional experience	Below 2 years	34 (50.0)	34 (50.0)	X^2^ = 6.746 *p* = 0.080
	2–5 years	18 (46.2)	21 (53.8)	
	5–8 years	15 (41.7)	21 (58.3)	
	Above 8 years	82 (61.7)	51 (38.3)	
Work schedule	Regular	93 (68.9)	42 (31.1)	**X^2^ = 23.628** ***p* < 0.001**
	Shift	56 (39.7)	85 (60.3)	
Presence of chronic disease	No	122 (55.5)	98 (44.5)	X^2^ = 0.942 *p* = 0.332
	Yes	27 (48.2)	29 (51.8)	
Mood status	Negative	98 (47.1)	110 (52.9)	**X^2^ = 16.041** ***p* < 0.001**
	Positive	51 (75.0)	17 (25.0)	
Perception of health status		6.59 ± 1.83	5.65 ± 2.02	t = −1.662 *p* = 0.098
Tobacco product use	Other	112 (57.1)	84 (42.9)	X^2^ = 2.714 *p* = 0.099
	Current user	37 (46.3)	43 (53.8)	
Alcohol use	No	142 (55.3)	115 (44.7)	X^2^ = 2.414 *p* = 0.120
	Yes	7 (36.8)	12 (63.2)	
Caffeinated beverages per day	1–2	57 (54.3)	48 (45.7)	X^2^ = 0.006 *p* = 0.937
	3 and above	92 (53.8)	79 (46.2)	
Mode of transportation to work	Walking	22 (47.8)	24 (52.2)	X^2^ = 1.008 *p* = 0.604
	Public transportation	46 (53.5)	40 (46.5)	
	Private vehicle	81 (56.3)	63 (43.7)	
Sleepiness after work	No	88 (75.2)	29 (24.8)	**X^2^ = 36.843** ***p* < 0.001**
	Yes	61 (38.4)	98 (61.6)	
Frequency of near- accident experience	None	65 (58.0)	47 (42.0)	X^2^ = 2.049 *p* = 0.359
	1–2	64 (53.3)	56 (46.7)	
	3 and above	20 (45.5)	24 (54.5)	
Type of near-accident (*n* = 164)	Home	42 (52.5)	38 (47.5)	X^2^ = 1.506 *p* = 0.471
	Traffic	37 (48.1)	40 (51.9)	
	Work	5 (71.4)	2 (28.6)	
Daily sleep duration	Below 6 h ^a^	34 (43.6)	44 (56.4)	**X^2^ = 6.458** ***p* = 0.040**
	6–8 h ^a,b^	85 (55.6)	68 (44.4)	
	Above 8 h ^b^	30 (66.7)	15 (33.3)	
Resignation intention		2.10 ± 1.31	7.23 ± 1.56	**t = −29.164** ***p* < 0.001**
DASS total score		17.91 ± 9.53	28.48 ± 11.60	**t = −8.177** ***p* < 0.001**
Depression		5.92 ± 3.77	9.50 ± 4.47	**t = −7.104** ***p* < 0.001**
Anxiety		5.55 ± 3.35	8.59 ± 3.84	**t = −7.032** ***p* < 0.001**
Stress		6.43 ± 3.23	10.37 ± 4.13	**t = −8.702** ***p* < 0.001**
Pittsburgh Sleep Quality Index (PSQI)		7.85 ± 3.08	10.52 ± 3.52	**t = −6.716** ***p* < 0.001**

χ^2^: Chi-square test; t: independent samples *t*-test.; ^a,b^ different superscript letters indicate statistically significant differences between groups. Bold values indicate significance.

**Table 7:** Multiple linear regression analysis of factors associated with PSQI total score.

	B	95% CI for B	SE	Std β	t
PSQI (R^2^ = 0.674; F = 31.337; *p* < 0.001)					
Constant	13.146	10.676	15.616	1.254	
DASS–Depression	0.057	−0.058	0.172	0.059	0.072
DASS–Anxiety	0.206	0.074	0.338	0.067	0.227
DASS–Stress	0.054	−0.072	0.181	0.064	0.064
Age	−0.010	−0.056	0.035	0.023	−0.022
Female	0.450	−0.149	1.049	0.304	0.060
Unmarried	0.003	−0.740	0.747	0.378	0.000
Presence of children: No	0.123	−0.694	0.941	0.415	0.017
BMI 25 below	0.365	−0.209	0.939	0.291	0.051
Chronic disease	0.343	−0.326	1.013	0.340	0.039
Nurse	−0.164	−0.784	0.457	0.315	−0.020
Non-administrative unit	−0.009	−0.896	0.879	0.451	−0.001
Shift work	2.295	1.663	2.927	0.321	0.324
Negative mood	0.117	−0.528	0.762	0.328	0.014
Sleepiness after work	0.397	−0.203	0.998	0.305	0.056

**Table 8:** Multiple linear regression analysis of factors associated with DASS total score.

	B	95% CI for B	SE	Std β	t
DASS score (R^2^ = 0.465; F = 14.898; *p* < 0.001)					
Constant	−0.964	−10.107	8.179	4.643	
Age	0.115	−0.081	0.310	0.099	0.075
Female	0.131	−2.423	2.685	1.297	0.005
Unmarried	2.705	−0.457	5.867	1.606	0.113
Presence of children: No	−0.458	−3.935	3.018	1.765	−0.019
Chronic disease	2.401	−0.462	5.264	1.454	0.082
Work experience <2 years	2.614	−0.459	5.686	1.560	0.096
Negative mood	4.146	1.439	6.852	1.374	0.152
Sleepy after work	3.313	0.779	5.847	1.287	0.139
No near-accident experience	−1.726	−3.969	0.518	1.139	−0.072
Job dissatisfaction	5.177	2.690	7.663	1.263	0.219
PSQI score	1.248	0.878	1.618	0.188	0.376
Shift work	−0.431	−3.049	2.188	1.330	−0.018

**Table 9:** Multivariable logistic regression analysis of factors associated with job dissatisfaction.

	OR (95% CI) *	*p*-Values
Age	0.96 (0.91–1.01)	0.094
Female	0.73 (0.35–1.53)	0.404
Unmarried	0.58 (0.28–1.17)	0.129
Chronic disease	1.62 (0.70–3.74)	0.256
BMI	0.95 (0.87–1.04)	0.276
Non-administrative unit	3.08 (0.84–11.27)	0.089
Shift work	2.02 (0.99–4.11)	0.052
Negative mood	1.86 (0.87–3.97)	0.111
Perceived health status	0.92 (0.77–1.10)	0.362
Tobacco Use	1.50 (0.76–2.99)	0.244
Sleepiness after work	1.85 (0.96–3.55)	0.065
PSQI score	1.07 (0.96–1.19)	0.245
DASS total score	1.07 (1.03–1.11)	**<0.001**

* OR values are presented with 95% confidence intervals. Model fit was assessed using the Hosmer–Lemeshow goodness-of-fit test, which was non-significant (χ^2^ = 2.844, df = 8, *p* = 0.944), indicating acceptable model fit. The overall correct classification rate of the logistic regression model was 76.7%. Bold values indicate significance.

## Data Availability

The data file has been uploaded to the submission system. The raw data supporting the conclusions of this article will be made available by the authors on request.

## Supplementary Materials

The following supporting information can be downloaded at: https://www.mdpi.com/article/10.3390/healthcare14152372/s1, Table S1: Bivariable screening and predictor selection for the multiple linear regression model of PSQI total score; Table S2: Bivariable screening and predictor selection for the multiple linear regression model of DASS total score; Table S3: Bivariable screening and predictor selection for the multivariable logistic regression model of job dissatisfaction; Table S4: Statistical mediation analysis of shift work and psychological distress through sleep quality.

## References

[1] Abate H., Letta S., Worku T., Tesfaye D., Amare E., Mechal A. (2023). Shiftwork sleep disorder and associated factors among nurses working at public hospitals in Harari Regional State and Dire Dawa Administration, Eastern Ethiopia: A cross-sectional study. BMC Nurs..

[2] Juliana N., Mohd Azmi N.A.S., Effendy N., Mohd Fahmi Teng N.I., Azmani S., Baharom N., Mohamad Yusuff A.S., Abu I.F. (2022). Exploring the associated factors of depression, anxiety, and stress among healthcare shift workers during the COVID-19 pandemic. Int. J. Environ. Res. Public Health.

[3] Geniş B., Coşar B., Taner M.E. (2020). Sağlık çalışanlarında ruhsal durumu etkileyen faktörler ve vardiyalı çalışma sisteminin etkileri. J. Psychiatr. Nurs..

[4] Parent-Thirion A., Biletta I., Cabrita J., Vargas Llave O., Vermeylen G., Wilczynska A., Wilkens M. (2017). Sixth European Working Conditions Survey—Overview Report, 2017 Update.

[5] Ferri P., Guadi M., Marcheselli L., Balduzzi S., Magnani D., Di Lorenzo R. (2016). The impact of shift work on the psychological and physical health of nurses in a general hospital: A comparison between rotating night shifts and day shifts. Risk Manag. Healthc. Policy.

[6] Batat H., Baniamer A.Z., Hamasha A.M., Abu Sahyoun A.M., AlSamhori J.F., Alsharqwi M.Z., Al-Aqtash M.J., Al-Qurneh M.K., Khalifeh A.H. (2024). The relationship between night shift work, sleep patterns, psychological well-being, and mental health among Jordanian healthcare workers. Arch. Environ. Occup. Health.

[7] Czyż-Szypenbejl K., Mędrzycka-Dąbrowska W. (2024). The impact of night work on the sleep and health of medical staff—A review of the latest scientific reports. J. Clin. Med..

[8] Li J., Cao D., Huang Y., Chen Z., Wang R., Dong Q., Wei Q., Liu L. (2022). Sleep duration and health outcomes: An umbrella review. Sleep Breath..

[9] Roman P., Perez-Cayuela I., Gil-Hernández E., Rodriguez-Arrastia M., Aparicio-Mota A., Ropero-Padilla C., Rueda-Ruzafa L. (2023). Influence of shift work on the health of nursing professionals. J. Pers. Med..

[10] Seong J., Son S., Min A. (2022). Effect of sleep on alertness at work among fixed night shift nurses: A prospective observational study. J. Adv. Nurs..

[11] Lanza G., DelRosso L.M., Ferri R. (2022). Sleep and homeostatic control of plasticity. Handb. Clin. Neurol..

[12] Radwan B., Yanez Touzet A., Hammami S., Chaudhury D. (2021). Prolonged exposure to social stress impairs homeostatic sleep regulation. Front. Neurosci..

[13] Qanash S., Alwafi H., Barasheed S., Bashnaini S., Andergiri R., Yaghmour L., Murad W., Shabrawishi M., Naser A.Y., Alsywid B. (2021). Impact of night shifts on sleeping patterns, psychosocial and physical well-being among healthcare professionals: A cross-sectional study in a tertiary hospital in Saudi Arabia. BMJ Open.

[14] Wyse C.A., Celis Morales C.A., Graham N., Fan Y., Ward J., Curtis A.M., Mackay D., Smith D.J., Bailey M.E.S., Biello S. (2017). Adverse metabolic and mental health outcomes associated with shiftwork in a population-based study of 277,168 workers in UK Biobank. Ann. Med..

[15] Medic G., Wille M., Hemels M.E.H. (2017). Short- and long-term health consequences of sleep disruption. Nat. Sci. Sleep.

[16] Alfonsi V., Scarpelli S., Gorgoni M., Pazzaglia M., Giannini A.M., De Gennaro L. (2021). Sleep-related problems in night shift nurses: Towards an individualized interventional practice. Front. Hum. Neurosci..

[17] Erren T.C., Morfeld P., Groß J.V., Wild U., Lewis P. (2019). IARC 2019: “Night shift work” is probably carcinogenic: What about disturbed chronobiology in all walks of life?. J. Occup. Med. Toxicol..

[18] Farha R.A., Alefishat E. (2018). Shift work and the risk of cardiovascular diseases and metabolic syndrome among Jordanian employees. Oman Med. J..

[19] Wendeu-Foyet M.G., Cénée S., Koudou Y., Trétarre B., Rébillard X., Cancel-Tassin G., Cussenot O., Boland A., Olaso R., Deleuze J. (2020). Circadian genes polymorphisms, night work and prostate cancer risk: Findings from the EPICAP study. Int. J. Cancer.

[20] Nehme P.A., Amaral F.G., Middleton B., Lowden A., Marqueze E., França-Junior I., Antunes J., Cipolla-Neto J., Skene D., Moreno C. (2019). Melatonin profiles during the third trimester of pregnancy and health status in the offspring among day and night workers: A case series. Neurobiol. Sleep Circadian Rhythm..

[21] Correia F.G.S., Ferreira M.J.M., Giatti L., Camelo L.V., Araújo L.F. (2020). Night work is related to higher global and central adiposity in Brazil: National Health Survey, 2013. Am. J. Ind. Med..

[22] Specht I.O., Hammer P.E.C., Flachs E.M., Begtrup L.M., Larsen A.D., Hougaard K.S., Hansen J., Hansen Å.M., Kolstad H.A., Rugulies R. (2019). Night work during pregnancy and preterm birth—A large register-based cohort study. PLoS ONE.

[23] Meléndez-Fernández O.H., Liu J.A., Nelson R.J. (2023). Circadian rhythms disrupted by light at night and mistimed food intake alter hormonal rhythms and metabolism. Int. J. Mol. Sci..

[24] Casas-Méndez C., Martín-Parrilla M.Á., Cáceres M.C., Montanero-Fernández J., López-Jurado C.F., Durán-Gómez N. (2026). Effects of night shifts on sleep, cognitive performance, and anxiety in emergency nurses: An observational within-subject study. J. Nurs. Manag..

[25] Querstret D., O’Brien K., Skene D.J., Maben J. (2020). Improving fatigue risk management in healthcare: A systematic scoping review of sleep-related/fatigue-management interventions for nurses and midwives. Int. J. Nurs. Stud..

[26] James S.M., Honn K.A., Gaddameedhi S., Van Dongen H.P.A. (2017). Shift work: Disrupted circadian rhythms and sleep—Implications for health and well-being. Curr. Sleep Med. Rep..

[27] Chen G.X., Fang Y., Guo F., Hanowski R.J. (2016). The influence of daily sleep patterns of commercial truck drivers on driving performance. Accid. Anal. Prev..

[28] Alreshidi S.M., Rayani A.M. (2023). The correlation between night shift work schedules, sleep quality, and depression symptoms. Neuropsychiatr. Dis. Treat..

[29] Li Y.X., Wang Y.C., Lv X.Y., Li R., Guan X.Y., Li L., Li J.L., Cao Y.J. (2022). Effects of factors related to shift work on depression and anxiety in nurses. Front. Public Health.

[30] Haile K.K., Asnakew S., Waja T., Kerbih H.B. (2019). Shift work sleep disorders and associated factors among nurses at federal government hospitals in Ethiopia: A cross-sectional study. BMJ Open.

[31] Brown J.P., Martin D., Nagaria Z., Verceles A.C., Jobe S.L., Wickwire E.M. (2020). Mental health consequences of shift work: An updated review. Curr. Psychiatry Rep..

[32] Cousin L., Roucoux G., Petit A.S., Baumann-Coblentz L., Torrente O.R., Cannafarina A., Chassany O., Duracinsky M., Carrieri P. (2022). Perceived stigma, substance use and self-medication in night-shift healthcare workers: A qualitative study. BMC Health Serv. Res..

[33] Buivydienė A., Rapolienė L., Truš M., Jakavonytė-Akstinienė A. (2025). Connections between job satisfaction and depression, anxiety, and stress among nurses. Front. Psychol..

[34] Quesada-Puga C., Izquierdo-Espin F.J., Membrive-Jiménez M.J., Aguayo-Estremera R., Cañadas-De La Fuente G.A., Romero-Béjar J.L., Gómez-Urquiza J.L. (2024). Job satisfaction and burnout syndrome among intensive-care unit nurses: A systematic review and meta-analysis. Intensive Crit. Care Nurs..

[35] Lovibond P.F., Lovibond S.H. (1995). The structure of negative emotional states: Comparison of the Depression Anxiety Stress Scales (DASS) with the Beck Depression and Anxiety Inventories. Behav. Res. Ther..

[36] Sarıçam H. (2018). The psychometric properties of Turkish version of Depression Anxiety Stress Scale-21 (DASS-21) in health control and clinical samples. J. Cogn. Behav. Psychother. Res..

[37] Buysse D.J., Reynolds C.F., Monk T.H., Berman S.R., Kupfer D.J. (1989). The Pittsburgh Sleep Quality Index: A new instrument for psychiatric practice and research. Psychiatry Res..

[38] Ağargün M.Y., Kara H., Anlar Ö. (1996). Pittsburgh Uyku Kalite İndeksi’nin geçerliği ve güvenirliği. Türk Psikiyatr. Derg..

[39] Hayes A.F. (2022). Introduction to Mediation, Moderation, and Conditional Process Analysis: A Regression-Based Approach.

[40] Nena E., Katsaouni M., Steiropoulos P., Theodorou E., Constantinidis T.C., Tripsianis G. (2018). Effect of shift work on sleep, health, and quality of life of health-care workers. Indian J. Occup. Environ. Med..

[41] Alshahrani S.M., Baqays A.A., Alenazi A.A., AlAngari A.M., AlHadi A.N. (2017). Impact of shift work on sleep and daytime performance among health care professionals. Saudi Med. J..

[42] Karhula K., Hakola T., Koskinen A., Ojajärvi A., Kivimäki M., Härmä M. (2018). Permanent night workers’ sleep and psychosocial factors in hospital work: A comparison to day and shift work. Chronobiol. Int..

[43] Lauren S., Chen Y., Friel C., Chang B.P., Shechter A. (2020). Free-living sleep, food intake, and physical activity in night and morning shift workers. J. Am. Coll. Nutr..

[44] Lin S.H., Liao W.C., Chen M.Y., Fan J.Y. (2014). The impact of shift work on nurses’ job stress, sleep quality and self-perceived health status. J. Nurs. Manag..

[45] Choi D.S., Kim S.H. (2022). Factors affecting occupational health of shift nurses: Focusing on job stress, health promotion behavior, resilience, and sleep disturbance. Saf. Health Work.

[46] Chang W.P., Chang Y.P. (2019). Relationship between job satisfaction and sleep quality of female shift-working nurses: Using shift type as moderator variable. Ind. Health.

[47] Malak M.Z., Al-Amer R.M., Abu Adas M.H. (2023). The influence of shift-work on perceived stress, sleep quality, and body mass index among emergency nurses. J. Hum. Behav. Soc. Environ..

[48] Chen X., Liu P., Lei G.F., Tong L., Wang H., Zhang X.Q. (2021). Sleep quality and the depression-anxiety-stress state of frontline nurses who perform nucleic acid sample collection during COVID-19: A cross-sectional study. Psychol. Res. Behav. Manag..

[49] Xi S., Gu Y., Guo H., Jin B., Guo F., Miao W., Zhang L. (2022). Sleep quality status, anxiety, and depression status of nurses in infectious disease department. Front. Psychol..

[50] Dai C., Qiu H., Huang Q., Hu P., Hong X., Tu J., Xie Q., Li H., Ren W., Ni S. (2019). The effect of night shift on sleep quality and depressive symptoms among Chinese nurses. Neuropsychiatr. Dis. Treat..

[51] Rodwell J., Fernando J. (2016). Managing work across shifts: Not all shifts are equal. J. Nurs. Scholarsh..

[52] Nirupama A.Y., Chellaiyan V.G., Qadeer A.S., Paulson W., Pandey S.P., Ravivarman G. (2024). Impact of rotational shiftwork-related sleep deprivation on quality of life and job satisfaction among healthcare professionals in Southern India from the SNORE study findings. Discov. Public Health.

[53] Sánchez-Sellero M.C., Sánchez-Sellero P., Fernández-Sánchez E. (2025). Effect of shift work on health and job satisfaction of European workers (EU-28): A cross-sectional and longitudinal study. Rev. Econ. Mund..

[54] Bingöl H., Güzel Özdemir P. (2023). Assessment of impulsivity, circadian preferences, and job satisfaction in nurses working with shift system. East. J. Med..

[55] Gebregziabher D., Berhanie E., Berihu H., Belstie A., Teklay G. (2020). The relationship between job satisfaction and turnover intention among nurses in Axum Comprehensive and Specialized Hospital, Tigray, Ethiopia. BMC Nurs..

[56] Li X., Wang J., He L., Hu Y., Li C., Xie Y., Wang N., Luo A., Lu Z. (2024). Turnover intention and influential factors among primary healthcare workers in Guangdong Province, China: A cross-sectional study. BMJ Open.

[57] O’Callaghan C., Sadath A. (2025). Exploring job satisfaction and turnover intentions among nurses: Insights from a cross-sectional study. Cogent Psychol..

[58] Xu K., Lei L., Guo Z., Liu X., Shi Y., Han G., Lin K., Cai W., Lu C., Li X. (2024). Turnover intention among healthcare workers in Shenzhen, China: The mediating effect of job satisfaction and work engagement. BMC Health Serv. Res..

[59] Alanmi B., Alharazi R., Almutary H. (2025). Night shift and occupational fatigue among nurses who work 12 hours in Jeddah. J. Educ. Health Promot..

[60] McElroy S.F., Olney A., Hunt C., Glennon C. (2020). Shift work and hospital employees: A descriptive multi-site study. Int. J. Nurs. Stud..

[61] Grøtting G., Øvergård K.I. (2023). The relation between routines for shiftwork scheduling and sickness absence at a Norwegian hospital: A cross-sectional study. Int. J. Nurs. Stud..

[62] Mortensen M., Torheim O.M., Berg G.V. (2026). Quick returns between shifts and nurse health: A scoping review. Nurs. Forum.

[63] Djupedal I.L.R., Harris A., Svensen E., Pallesen S., Waage S., Nielsen M.B., Sunde E., Bjorvatn B., Holmelid Ø., Vedaa Ø. (2024). Effects of a work schedule with abated quick returns on insomnia, sleepiness, and work-related fatigue: Results from a large-scale cluster randomized controlled trial. Sleep.

[64] Blasche G., Bauböck V.M., Haluza D. (2017). Work-related self-assessed fatigue and recovery among nurses. Int. Arch. Occup. Environ. Health.

[65] Haluza D., Schmidt V.M., Blasche G. (2019). Time course of recovery after two successive night shifts: A diary study among Austrian nurses. J. Nurs. Manag..

[66] Qtait M., Al Ali M.F., Jaradat Y. (2025). The impact of rotating shift work on nurse burnout: A systematic review of contributing factors and organisational strategies. SAGE Open Nurs..

[67] Epstein M., Arakelian E., Tucker P., Dahlgren A. (2023). Managing sustainable working hours within participatory working time scheduling for nurses and assistant nurses: A qualitative interview study with managers and staffing assistants. J. Nurs. Manag..

[68] Brants P., Hanefeld S., Jacobs A.S., Hüttl J., Hanika S., Lücker K., Vey D. (2026). The impact of working time conditions on nurses’ private life: A cross-sectional study on spillover effects and structural factors for recovery. BMC Nurs..

[69] Boskma A., van der Braak K., Hooft L., Oerbekke M.S., Franx A., van der Laan M. (2025). Effectiveness of organization-directed interventions on healthcare professionals’ well-being: A systematic review. eClinicalMedicine.

[70] van Elk F., Lammers-van der Holst H.M., Robroek S.J.W., Burdorf A., Oude Hengel K.M. (2024). Effects and implementation of an intervention to improve sleep, fatigue and recovery among healthcare workers with night shifts: A pre- and post-test study. Int. J. Nurs. Stud..

[71] Aust B., Leduc C., Cresswell-Smith J., O’brien C., Rugulies R., Leduc M., Ni Dhalaigh D., Dushaj A., Fanaj N., Guinart D. (2024). The effects of different types of organisational workplace mental health interventions on mental health and wellbeing in healthcare workers: A systematic review. Int. Arch. Occup. Environ. Health.

[72] Gifkins J., Johnston A., Loudoun R., Troth A. (2020). Fatigue and recovery in shiftworking nurses: A scoping literature review. Int. J. Nurs. Stud..

