# Supplementary material for: Shift Work and Psychological Distress Among Healthcare Workers: The Mediating Role of Sleep Quality and the Association with Job Dissatisfaction
Source: Healthcare (Basel). 2026 Aug 3;14(15):2372. doi: 10.3390/healthcare14152372 (PMC13465422; doi:10.3390/healthcare14152372)
Supplement: Supplementary file 1 [file healthcare-14-02372-s001.zip › healthcare-4440467-supplementary.pdf]

# Supplementary Tables

Variables with $p < 0.25$ in bivariable analyses were considered candidate predictors. Variables selected a priori on theoretical or clinical grounds could also be retained irrespective of their bivariable p-values. Final inclusion decisions are shown below.

## Table S1. Bivariable screening and predictor selection for the multiple linear regression model of PSQI total score

| Candidate predictor | Corresponding bivariable variable | Bivariable p-value | Final model status |
|---|---|---|---|
| DASS–Depression | Depression score | <0.001 | Included; met p < 0.25 criterion |
| DASS–Anxiety | Anxiety score | <0.001 | Included; met p < 0.25 criterion |
| DASS–Stress | Stress score | <0.001 | Included; met p < 0.25 criterion |
| Age | Age group | 0.068 | Included; met p < 0.25 criterion |
| Female | Sex | 0.011 | Included; met p < 0.25 criterion |
| Unmarried | Marital status | 0.013 | Included; met p < 0.25 criterion |
| No children | Presence of children | 0.025 | Included; met p < 0.25 criterion |
| BMI <25 | BMI category | 0.028 | Included; met p < 0.25 criterion |
| Chronic disease | Presence of chronic disease | 0.025 | Included; met p < 0.25 criterion |
| Nurse | Occupation | 0.040 | Included; met p < 0.25 criterion |
| Non-administrative unit | Work unit | 0.002 | Included; met p < 0.25 criterion |
| Shift work | Work schedule | <0.001 | Included; met p < 0.25 criterion |
| Negative mood | Mood status | <0.001 | Included; met p < 0.25 criterion |
| Sleepiness after work | Sleepiness after work | <0.001 | Included; met p < 0.25 criterion |
| No near-accident experience | Frequency of near-accident experience | 0.001 | Included; met p < 0.25 criterion |
| Sleep duration | Daily sleep duration | <0.001 | Included; met p < 0.25 criterion |
| Job dissatisfaction | Job satisfaction | <0.001 | Included; met p < 0.25 criterion |

All listed variables were entered simultaneously using the enter method.

## Table S2. Bivariable screening and predictor selection for the multiple linear regression model of DASS total score

| Candidate predictor | Corresponding bivariable variable | Bivariable p-value | Final model status and selection basis |
|---|---|---|---|
| Age | Age group | 0.290 | Included a priori because of theoretical and clinical relevance |
| Female | Sex | 0.136 | Included; met p < 0.25 criterion |
| Unmarried | Marital status | <0.001 | Included; met p < 0.25 criterion |
| No children | Presence of children | 0.005 | Included; met p < 0.25 criterion |
| Chronic disease | Presence of chronic disease | 0.049 | Included; met p < 0.25 criterion |
| Work experience <2 years | Years of professional experience | 0.154 | Included; met p < 0.25 criterion |
| Negative mood | Mood status | <0.001 | Included; met p < 0.25 criterion |
| Sleepiness after work | Sleepiness after work | <0.001 | Included; met p < 0.25 criterion |
| No near-accident experience | Frequency of near-accident experience | <0.001 | Included; met p < 0.25 criterion |
| Job dissatisfaction | Job satisfaction | <0.001 | Included; met p < 0.25 criterion |
| PSQI score | PSQI total score/categories | <0.001 | Included as the continuous sleep-quality measure |
| Shift work | Work schedule | <0.001 | Included; met p < 0.25 criterion |
| Non-administrative unit | Work unit | 0.040 | Included; met p < 0.25 criterion |
| Tobacco product use | Tobacco product use | 0.156 | Included; met p < 0.25 criterion |
| ≥3 caffeinated beverages/day | Caffeinated beverages per day | 0.148 | Included; met p < 0.25 criterion |
| Type of near-accident | Type of near-accident (N = 164) | 0.176 | Not retained because it was available only for participants reporting a near-accident; inclusion would reduce the analytic sample |

Age was retained a priori despite p = 0.290. The remaining variables included in the final model met the p < 0.25 screening criterion.

## Table S3. Bivariable screening and predictor selection for the multivariable logistic regression model of job dissatisfaction

| Candidate predictor | Bivariable p-value | Final model status | Selection basis |
|---|---|---|---|
| Age | 0.003 | Included | Met p < 0.25 criterion |
| Female sex | 0.880 | Included a priori | Retained as a theoretically and clinically relevant covariate |
| Unmarried | 0.010 | Included | Met p < 0.25 criterion |
| Chronic disease | 0.332 | Included a priori | Retained as a theoretically and clinically relevant covariate |
| BMI | 0.017 | Included | Met p < 0.25 criterion |
| Non-administrative unit | <0.001 | Included | Met p < 0.25 criterion |
| Shift work | <0.001 | Included | Met p < 0.25 criterion |
| Negative mood | <0.001 | Included | Met p < 0.25 criterion |
| Perceived health status | 0.098 | Included | Met p < 0.25 criterion |
| Tobacco use | 0.099 | Included | Met p < 0.25 criterion |
| Sleepiness after work | <0.001 | Included | Met p < 0.25 criterion |
| PSQI score | <0.001 | Included | Met p < 0.25 criterion |
| DASS total score | <0.001 | Included | Selected as the primary psychological-distress measure |
| Presence of children | 0.014 | Not retained | Not retained to limit redundancy with age and marital status |
| Years of professional experience | 0.080 | Not retained | Not retained to limit redundancy with age |
| Alcohol use | 0.120 | Not retained | Only 19 participants reported alcohol use; exclusion reduced the risk of unstable estimates |
| Daily sleep duration | 0.040 | Not retained | PSQI was retained as the principal sleep-related predictor to limit construct overlap |
| Resignation intention | <0.001 | Not retained | Conceptually proximal to job dissatisfaction and potentially downstream of it |
| DASS–Depression | <0.001 | Not retained | DASS total score was retained to avoid redundancy among psychological-distress measures |
| DASS–Anxiety | <0.001 | Not retained | DASS total score was retained to avoid redundancy among psychological-distress measures |
| DASS–Stress | <0.001 | Not retained | DASS total score was retained to avoid redundancy among psychological-distress measures |

Predictors retained in the final model were entered simultaneously using the enter method. Female sex and chronic disease were retained a priori despite not meeting the $p < 0.25$ screening criterion.

## Table S4. Statistical mediation analysis of shift work and psychological distress through sleep quality

| Path/effect | Description | B | SE/BootSE | t | p | 95% CI |
|---|---|---|---|---|---|---|
| Path a | Shift work → sleep quality | 2.52 | — | — | <0.001 | — |
| Path b | Sleep quality → psychological distress | 1.78 | — | — | <0.001 | — |
| Total effect, c | Shift work → psychological distress | 4.62 | 1.43 | 3.22 | 0.001 | 1.79 to 7.44 |
| Direct effect, c′ | Shift work → psychological distress, adjusted for sleep quality | 0.12 | 1.31 | 0.09 | 0.927 | −2.46 to 2.71 |
| Indirect effect, a × b | Shift work → sleep quality → psychological distress | 4.50 | 0.87[1] | — | — | 2.87 to 6.26[1] |

[1] Bootstrap standard error and 95% bootstrap confidence interval based on 5000 resamples.

Note: Unstandardized coefficients (B) are presented. The indirect association was statistically significant because its 95% bootstrap confidence interval did not include zero. The model was adjusted for age, sex, marital status, and presence of chronic disease. Because the data were cross-sectional, the mediation findings do not establish temporal or causal mediation. An em dash indicates that the statistic was not reported or was not applicable.
